# Pulmonary Granulomas and Mycobacterial Infection: Concordance between the Results of Special Stains Performed on Lung Tissue Sections and Tissue Cultures

**DOI:** 10.3390/diseases10040096

**Published:** 2022-11-01

**Authors:** Hisham F. Bahmad, Roshanak Azimi, Ekim Kilinc, Claudio Tuda, Cristina Vincentelli

**Affiliations:** 1 Department of Pathology and Laboratory Medicine, Mount Sinai Medical Center, Miami Beach, FL 33140, USA; 2 Department of Internal Medicine, Mount Sinai Medical Center, Miami Beach, FL 33140, USA; 3 Department of Translational Medicine, Herbert Wertheim College of Medicine, Florida International University, Miami, FL 33199, USA

**Keywords:** pulmonary granulomas, mycobacteria, FITE, Kinyoun acid-fast, culture, concordance

## Abstract

Background: The most common cause of infectious pulmonary granulomas worldwide is *Mycobacterium tuberculosis*. The diagnosis is based on clinical presentation, histopathologic findings, detection of acid-fast bacilli (AFB) in tissue or sputum using special stains, and/or isolation of mycobacteria in cultures or via PCR-based methods. Different studies have shown that high levels of discrepancy exist between these diagnostic approaches in lung tissue specimens. Objective: To assess the degree of concordance between the results of special stains and cultures on lung tissue specimens in the diagnosis of mycobacterial infections. Methodology: Eighty-seven patients with a diagnosis of granulomas (necrotizing and non-necrotizing) on lung tissue specimens were identified. Cohen’s kappa was used to measure the general concordance between the results of the histopathological examination (special stains) and bacteriological tissue cultures. Results: With Kinyoun acid-fast stains, 8/48 (16.7%) cases were positive for AFB. With FITE stains, 10/57 (17.5%) cases were positive for AFB. There was strong agreement between Kinyoun acid-fast and FITE stains (Kappa = 0.806; *p*-value < 0.001). Tissue cultures were performed on 38/87 cases (43.7%), and 10/38 (26.3%) of the cultures were positive for mycobacteria. There was no concordance between Kinyoun acid-fast stains or FITE stains and tissue cultures results. Conclusion: Our observations represent an initial step in the process of reviewing the two methods used at our institution to diagnose mycobacterial infections on lung tissue specimens and highlight the need of incorporating more advanced diagnostic methods such as PCR to confirm mycobacterial infections and improve patient management. Importantly, species-level identification of mycobacteria is necessary to guide treatment.

## 1. Introduction

Granulomas are organized aggregates of epithelioid macrophages [1] that are thought to form as a result of a complex innate and adaptive cellular immune process [2]. They may be non-necrotizing, necrotizing, or suppurative. Different infectious organisms may cause granuloma formation including mycobacteria (usually necrotizing granulomas), fungi (such as histoplasmosis, cryptococcosis, blastomycosis, and coccidioidomycosis), parasites (schistosomiasis and leishmaniasis), and other bacteria or rarely viruses [3]. There are also noninfectious causes of granulomas including but not limited to autoimmune diseases, foreign bodies, and neoplasms. Nevertheless, the most common cause of infectious pulmonary granulomas worldwide is *Mycobacterium tuberculosis* [4]. The latter can invade different organs in the body, mainly the lungs causing pneumonia [5]. Clinically, it can present either as acute tuberculous pneumonia or as asymptomatic incidental pulmonary nodule on imaging which turns to be granulomatous. In fact, it is fairly uncommon for pulmonary mycobacterial infections to manifest as acute, rapidly progressive pneumonia [6].

The diagnosis of tuberculosis may be challenging and is occasionally made at our institution on lung specimens resected for lesions that are suspicious for malignancy on imaging. In those cases, the patient usually undergoes a positron emission tomography/computed tomography (PET/CT) scan where a lesion/nodule lights up demonstrating increased uptake and raising suspicion for malignancy [7]. Surgical resection is sought, and the specimen is sent for intraoperative consultation. In up to 20% of cases, pathology is benign [8], majority of which shows granulomas ruling out a neoplastic process. When enough tissue is available for histopathological examination besides tissue cultures, the diagnosis is based on the presence of granulomas (usually necrotizing), the detection of organisms with the use of special stains for AFB or with immunohistochemistry [9], and/or isolation of *M. tuberculosis* in tissue cultures or via PCR-based methods [10]. Studies have shown that tuberculosis and primary lung carcinomas have similar glucose metabolic activity [11,12], which is best explained by the excessive activity of macrophages and neutrophils in the tissues [13].

When pulmonary tuberculosis infection is suspected clinically and on imaging studies, the 2017 ATS/IDSA/CDC tuberculosis diagnosis guidelines recommend that post-bronchoscopy sputum specimens be obtained [14]. The recommendation is that AFB smear microscopy be performed in all patients suspected of having pulmonary tuberculosis. Additionally, it is suggested to perform both liquid and solid mycobacterial cultures for every specimen obtained, and to do a diagnostic nucleic acid amplification test (NAAT) [14]. However, as mentioned above, in our pathology practice is not infrequent to encounter mycobacterial infections in patients who clinically and radiologically appeared to harbor a pulmonary neoplasm. Therefore, none of our patients had sputum specimen available to assess for the diagnosis of mycobacteria or other infectious agents.

Different studies and our personal experience have shown that high levels of discrepancy exist between the aforementioned diagnostic approaches. Therefore, it is crucial to assess the degree of concordance between the results of the two methods routinely used at our institution for the diagnosis of mycobacterial infections in lung tissue specimens: histopathological examination using special stains and tissue cultures. Our goal is to develop a quality improvement project that will incorporate an improved diagnostic protocol, and that will later translate into structured guidelines for patient management.

## 2. Materials and Methods

### 2.1. Study Design, Setting, and Objectives

The pathological database of pulmonary granuloma specimens from patients who underwent surgical resections/biopsies during a seventeen-year period, from 1 January 2005 to 31 July 2022, at Mount Sinai Medical Center (Miami Beach, FL, USA) was reviewed. This study represents a single-center retrospective cohort analysis of patients treated at an academic medical center. Medical chart review was performed, and patient information were collected. There was no direct physical or mental risk to the patients in the study. Objectives of our study included: (1) collection of the clinical and pathological data of patients with a diagnosis of pulmonary granulomas, (2) histopathological examination of lung tissue specimens with granulomas and assessment of the results of special stains (Kinyoun acid-fast and FITE), (3) examination of the results of tissue cultures and mycobacterium polymerase chain reaction (PCR) results if available, and lastly, (4) exploring the concordance between the results of special stains performed on lung tissue sections and tissue cultures for the diagnosis of mycobacterial infection.

### 2.2. Ethical Considerations

Approval of the Institutional Review Board (IRB) of Mount Sinai Medical Center of Florida was granted prior to commencement of the study. All protocols followed in our retrospective cohort study were performed in accordance with guidelines and regulations of The Code of Ethics of the World Medical Association (Declaration of Helsinki). The study was done in a manner that ensures confidentiality of patients. Chart review was carried out by CITI (Collaborative Institutional Training Initiative) certified resident physicians. All data collected were de-identified and stored at the principal investigator’s office. Data were stored in a computer using a number to identify each patient in place of their name (de-identification). A database form was completed on each patient using a number to identify the patient. The data was stored on one computer in the principal investigator’s office. The computer was password protected, and the database itself was password protected as well. The only members of the research study team with access were the principal investigators and pathology residents.

### 2.3. Patient Selection

Inclusion criteria included patients who underwent biopsies or surgical resections of the lung with a diagnosis of granulomas during the time-period specified. Only patients with unequivocal diagnoses were included. Exclusion criteria included cases with confirmed fungal organisms on special stains or tissue cultures. The patients included had biopsies or surgical resections of lung for lesions on imaging that were suspicious for malignancy and none of the patients suffered from signs or symptoms of mycobacterial infection initially. That being said, Quantiferon Gold test was not available to rule in or out a TB infection.

### 2.4. Tissue Cultures and Stains

Thirty-eight patients had fresh lung tissue specimens submitted for culture. In brief, tissues were obtained at the time of resection or biopsy. Standard conventional methods for isolation of mycobacteria were used. In brief, an AFB smear is made on each specimen. After processing and decontamination, every specimen was inoculated into a U slant and the BACTEC mycobacteria growth indicator tube (MGIT) 320 was used to detect growth. The BACTEC MGIT 320 instrument is used detect growth. MGIT tubes inoculated with patient’s specimen were entered into the BACTEC MGIT 320 system and were continuously incubated at 37 °C and monitored every 60 min for increasing fluorescence. Analysis of the fluorescence is used to determine if the tube in the instrument is positive. The Lowenstein Jensen (LJ) slant was also inoculated with the processed patients’ specimens. Once the MGIT turns positive, mycobacterial identification is pursued based on the following:

- If positive within 7 days (rapid grower), an isolate was sent to a reference lab for full identification and susceptibilities.
- If positive after day 7 (slow grower/possible *M. tuberculosis* or *M. avium complex*) DNA hybridization studies are performed for *M. tuberculosis* and *M. avium complex* by the GenProbe method.
- If negative for both, the following is reported: “Mycobacterium species, not *M. tuberculosis*, not *M. avium complex*”. (Since susceptibilities are not performed in house, the specimens are sent out to Reference Lab for further classification).

Kinyoun acid-fast and FITE special stains were performed using the BenchMark Special Stains AFB Staining Kit (Ventana Medical Systems, Inc., Tucson, Arizona, USA.; Catalog Number: 08432503001).

### 2.5. Statistical Analysis

Data retrieved from medical charts and pathology reports of patients were entered into a Microsoft Excel spreadsheet which was designed specifically for this study. The statistical analyses, data management, and cleaning were executed using the SPSS (IBM Corp., Released 2019, SPSS Statistics for Windows Version 26.0, Armonk, NY, USA). Descriptive statistics were reported as frequencies and percentages for categorical variables and as means (±) standard deviation (SD) for continuous ones. Cohen’s Kappa Statistic was used to measure the agreement between categorical variables. Specifically, Kappa was used to measure the reliability of the diagnosis and general concordance between the results of special stains and bacteriological tissue cultures. *p* values less than 0.05 were considered statistically significant.

## 3. Results

From our pathology database at Mount Sinai Medical Center of Florida, a total of 87 cases of pulmonary granulomas were identified between 1 January 2005, and 31 July 2022, of which 39 (44.8%) were females and 48 (55.2%) were males. The clinicopathological characteristics of patients are summarized in Table 1. The mean age of our patient cohort was 67.29 ± 12.76 years. Twenty-eight patients (32.2%) underwent lobectomies, 36 (41.4%) underwent wedge resections, and 23 (26.4%) underwent biopsies. Of the total 87 cases included, 62 (71.3%) were from the right side and 25 (28.7%) were from the left side. Twenty-six cases (30.0%) were from the right upper lobe (RUL), thirteen cases (14.9%) were from the right middle lobe (RML), twelve (13.8%) were from the right lower lobe (RLL), seventeen (19.5%) were from the left upper lobe, eight (9.2%) were from the left lower lobe, and eleven (12.6%) cases had unspecified sites. A diagnosis of necrotizing granuloma was present in 54 patients (62.1%) and non-necrotizing granuloma in 33 patients (37.9%) (Table 1).

Kinyoun acid-fast stains were performed on 48/87 (55.2%) cases. FITE stains were performed on 57/87 (65.5%) cases. With Kinyoun acid-fast stains, 8/48 (16.7%) cases were positive for AFB (Table 1). With FITE stains, 10/57 (17.5%) cases were positive for AFB (Table 1). There was strong agreement between Kinyoun acid-fast and FITE stains: Kappa = 0.806 (Table 2; *p*-value < 0.001). Specifically, 36 of 43 patients who had both Kinyoun acid-fast and FITE stains performed on them, demonstrated negative Kinyoun acid-fast stains and negative FITE stains as well, whereas 5 of those 43 patients showed positive Kinyoun acid-fast and FITE stains. Interestingly, disagreement between the two stains was found only in two patients: one patient had a positive Kinyoun acid-fast stain but negative FITE, and another patient had a positive FITE but negative Kinyoun acid-fast stain (Table 2).

Tissue cultures were performed on 38/87 cases (43.7%), and 10/38 (26.3%) of the cultures were positive for mycobacteria (Table 1). There was no concordance between Kinyoun acid-fast stains or FITE stains and tissue cultures results (Table 3 and Table 4). In Table 3, our results showed that the majority of cases with positive Kinyoun acid-fast stain (3/4; 75.0%) had negative tissue cultures. In addition, 4/19 (21.1%) patients with negative Kinyoun acid-fast had positive tissue cultures. Similarly, with FITE stain, half of the cases with positive stain (3/6; 50.0%) had negative tissue cultures and 4/22 (18.2%) patients with negative FITE stains had positive tissue cultures (Table 4).

**Figure 1:**
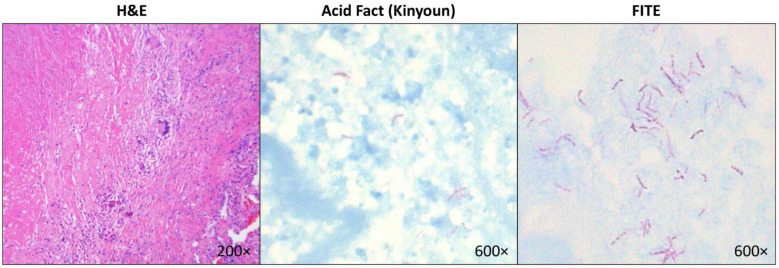
Representative images of a lung tissue section with necrotizing granuloma stained with Hematoxylin & Eosin (left panel) and mycobacteria identified on Kinyoun acid-fast stain (middle panel) and FITE stain (right panel) in a patient who grew *Mycobacterium kansasii*, which was confirmed by PCR.

Among the ten patients with positive lung tissue cultures for mycobacteria (Table 5), four had non-necrotizing granulomas (growing *M. tuberculosis*, *M. fortuitum-chelonei*, Mycobacterium avium complex, and *M. kansasii*) and six had necrotizing granulomas (growing *M. abscessus*, Mycobacterium avium complex, and *M. kansasii*). Four of the patients with positive tissue cultures had both negative Kinyoun acid-fast and FITE stains. Two patients had positive FITE but negative Kinyoun acid-fast stains and grew *M. abscessus* and *M. kansasii*. Only in one patient demonstrating positive FITE and Kinyoun acid-fast stains, tissue cultures grew *M. kansasii* (Figure 1). This patient also had a positive PCR result confirming mycobacterial infection.

## 4. Discussion

The histopathologic identification of granulomas is helpful in predicting the diagnostic etiology of the disease [15]. Different patterns can be recognized, including necrotizing, non-necrotizing, and suppurative granulomas. The organisms that are most commonly found in pulmonary granulomas are mycobacteria and fungi [16]. While *Mycobacterium tuberculosis* is expected to be frequently isolated in lung tissues of patients residing in developing countries, non-tuberculous mycobacteria (NTM) are often cultured from granulomas in the United States and other developed countries [17]. A definitive diagnosis of mycobacterial granulomas can often be made with ancillary testing such as cultures, special stains, and molecular diagnostics. Staining approaches are usually cheap and simple making them the initial approach for screening or diagnosing mycobacterial infections. However, they only have good sensitivity in active cases of mycobacterial infection with high load of bacteria. Though, they still can be a good choice in low-income developing countries with no access to more expensive testing tools such as PCR or culture since the number of active mycobacterial infection cases are usually higher compared to developed countries where most cases have low bacterial load rendering less sensitive results and poor correlation with culture or PCR.

A study by Ulbright and Katzenstein showed that around 25% of necrotizing granulomas remain unexplained although the majority are infectious in etiology [18]. The differential diagnosis of necrotizing granulomas includes besides mycobacterial infections, Wegner’s Granulomatosis (WG), aspiration pneumonia, rheumatoid nodule, necrotizing sarcoid granulomatosis (NSG), infarction, and lymphomatoid granulomatosis [8].

The granulomas of *M. tuberculosis* are typically necrotizing but may be non-necrotizing or a mix of both types [19]. When a mycobacterial infection is suspected based on the histopathologic findings, special stains for AFB are usually ordered, such as Kinyoun acid-fast and FITE. Kinyoun uses carbol-fuchsin as a primary stain, followed by decolorization with an acid-alcohol solution and methylene blue as a counterstain. FITE uses peanut oil mixed with xylene in the deparaffinization. Although the commonly used method is the Kinyoun’s method, FITE is just a modification of this stain that has a weaker acid for supposedly more delicate *M. leprae bacilli*. Immunohistochemistry for mycobacteria is more specific but has limitations, including lack of widespread availability [20]. In our study, which spanned a period of 17 years, 87 surgical lung tissue specimens with granulomas (necrotizing and non-necrotizing) were identified in our database. Tissue culture results were available in 38 of these 87 cases, in which 10/38 (26.3%) were positive for mycobacteria. Non-tuberculous mycobacteria like MAC, *M. kanasii*, *M. fortuitum*, and *M. abcessus* were isolated. A subset of these were negative for Kinyoun acid-fast and FITE stains.

Kinyoun acid-fast stains were performed on 48 specimens, and 8/41 (16.7%) cases were positive for AFB. FITE stains were performed on 57 specimens and 10/57 (17.5%) cases were positive for AFB. There was strong agreement between Kinyoun acid-fast and FITE stains, but there was a discrepancy between all stains and cultures, as shown in other studies [21]. At our institution, we do not attempt speciation of acid-fast bacilli based on the organism’s morphology on special stains performed on tissue sections.

It is important to note that most of the published literature on identification of mycobacteria by using microscopic morphologic features is based on smears made from microbiologic cultures rather than formalin-fixed, paraffin-embedded (FFPE) histologic material [2]. In fact, no data exist to show the accuracy of mycobacterial speciation by pathologists blinded to culture results [4]. Even in smears made from microbiologic cultures, the morphologic features such as cording, beading, or size, although associated with certain species, do not allow accurate or definitive speciation of mycobacteria [22].

Currently, the only definitive methods of mycobacterial speciation are microbiologic culture and molecular techniques, such as the polymerase chain reaction (PCR) [23]. When results of special histologic stains are positive, but those of cultures are negative, or when biopsied tissue is not submitted for culture, PCR is the only means of determining the species of the organism. Not infrequently, pathologists face a problem when mycobacteria are identified in tissue sections with the use of special stains, but speciation is impossible due to the lack of availability of fresh tissue for cultures, or because of negative culture results [4]. To overcome this issue, PCR and other molecular methods for detecting and speciating mycobacteria in FFPE tissue have been developed [24]. PCR is as sensitive as microbiologic cultures for detecting mycobacteria in FFPE specimens and is more sensitive than the conventional special stains [25]. In addition, it is also possible to determine the species of mycobacteria with PCR-based methods. Currently though, PCR for detection and speciation of mycobacteria in FFPE tissue remains restricted to a few reference laboratories.

Our study has some limitations. First, both special stains for AFB were not consistently ordered on all lung specimens with granulomas, and tissue cultures were only performed on specimens received for intraoperative consultation. Additionally, the sensitivity of special stains for organism detection could have been limited by the number of tissue blocks stained in each case, since in most cases stains were performed in a single tissue block even when granulomas were present in more than one block. In addition, an important limitation is the yield of cultures in pulmonary granulomas, which is likely dependent on the extent of infection, immune competence of the host, and presence or absence of viable organism within the specimen.

## 5. Conclusions

In our study, we demonstrated that there was no concordance between the results of special stains for AFB or FITE and the corresponding tissue cultures in the diagnosis of mycobacterial infections in lung resection specimens. These observations represent an initial step in the process of reviewing the two methods routinely used in our institution to diagnose mycobacterial infections on lung resection specimens with granulomas. Incorporating an improved diagnostic protocol and methods including PCR is essential to confirm mycobacterial infections and improve patient care management. Importantly, species-level identification of mycobacteria is necessary to guide treatment.

## Figures and Tables

**Table 1:** Clinicopathological characteristics of patients with pulmonary granulomas (87 patients).

Clinicopathological Variables	Number of Patients *n* (%)
Age (years)—Mean ± SD (*n* = 87)	67.29 ± 12.76
Age (years) (*n* = 87)	
<60	20 (23.0)
60–70	24 (27.6)
70–80	35 (40.2)
≥80	8 (9.2)
Gender (*n* = 87)	
Female	39 (44.8)
Male	48 (55.2)
Procedure (*n* = 87)	
Lobectomy	28 (32.2)
Biopsy	23 (26.4)
Wedge	36 (41.4)
Site (Lobe) (*n* = 87)	
RUL	26 (30.0)
RML	13 (14.9)
RLL	12 (13.8)
LUL	17 (19.5)
LLL	8 (9.2)
Unspecified	11 (12.6)
Side (*n* = 87)	
Right	62 (71.3)
Left	25 (28.7)
Diagnosis (*n* = 87)	
Necrotizing granuloma	54 (62.1)
Non-necrotizing granuloma	33 (37.9)
Kinyoun acid-fast (*n* = 48)	
Positive	8 (16.7)
Negative	40 (83.3)
FITE (*n* = 57)	
Positive	10 (17.5)
Negative	47 (82.5)
Lung tissue cultures (*n* = 38)	
Positive	10 (26.3)
Negative	28 (73.7)
Mycobacterium PCR (*n* = 8)	
Positive	2 (25.0)
Negative	6 (75.0)

**Abbreviations:** LLL: left lower lobe; LUL: left upper lobe; PCR: polymerase chain reaction; RLL: right lower lobe; RML: right middle lobe; RUL: right upper lobe; SD: standard deviation.

**Table 2:** Concordance in positivity of results between Kinyoun acid-fast and FITE stains performed on lung tissue sections (43 patients).

		Kinyoun Acid-Fast Stain			Kappa (*p*-Value)
		Negative	Positive	Total	
FITE stain	Negative	36 (97.3%)	1 (16.7%)	37	0.806 (<0.001)
	Positive	1 (2.7%)	5 (83.3%)	6	
	Total	37	6	43	

**Table 3:** Concordance in positivity of results between Kinyoun acid-fast stain performed on lung tissue sections and the corresponding tissue cultures (23 patients).

		Kinyoun Acid-Fast Stain			Kappa (*p*-Value)
		Negative	Positive	Total	
Tissue cultures	Negative	15 (78.9%)	3 (75.0%)	18	0
	Positive	4 (21.1%)	1 (25.0%)	5	
	Total	19	4	23	

**Table 4:** Concordance in positivity of results between FITE stain performed on lung tissue sections and the corresponding tissue cultures (28 patients).

		FITE Stain			Kappa (*p*-Value)
		Negative	Positive	Total	
Tissue cultures	Negative	18 (81.8%)	3 (50.0%)	21	0
	Positive	4 (18.2%)	3 (50.0%)	7	
	Total	22	6	28	

**Table 5:** Cases with positive lung tissue cultures for mycobacteria (10 patients).

Age (Years)	Gender	Lobe	Granuloma	Kinyoun Acid-Fast	FITE	*Mycobacterium* Organism	PCR	Follow Up
56	F	RUL	Necrotizing		+	*M. abscessus*	N/A	Loss of follow up
68	M	LUL	Necrotizing	-	-	MAC	N/A	Loss of follow up
62	M	-	Non-necrotizing			*M. tuberculosis*	N/A	History of TB treated before
77	F	RUL	Non-necrotizing			*M. fortuitum—chelonei*	N/A	Loss of follow up
42	M	RML	Non-necrotizing	-	-	MAC	N/A	Loss of follow up
82	F	RML	Necrotizing			MAC	N/A	No treatment
72	M	RLL	Necrotizing	-	-	MAC	N/A	Triple therapy for MAC for 2 months, repeated biopsy negative
70	F	RUL	Necrotizing	+	+	*M. kansasii*	+ve	Loss of follow up
76	F	LLL	Non-necrotizing	-	-	*M. kansasii*	N/A	No treatment
75	F	RUL	Necrotizing	-	+	*M. kansasii*	N/A	No treatment

**Abbreviations:** LLL: left lower lobe; LUL: left upper lobe; F: female; MAC: Mycobacterium avium complex; PCR: polymerase chain reaction; RLL: right lower lobe; RML: right middle lobe; RUL: right upper lobe; TB: tuberculosis.

## Data Availability

Not applicable.

## References

[1] Pagán A.J., Ramakrishnan L. (2018). The Formation and Function of Granulomas. Annu. Rev. Immunol..

[2] Guarner J. (2012). Detection of microorganisms in granulomas that have been formalin-fixed: Review of the literature regarding use of molecular methods. Scientifica.

[3] Mukhopadhyay S. (2011). Role of histology in the diagnosis of infectious causes of granulomatous lung disease. Curr. Opin. Pulm. Med..

[4] Mukhopadhyay S., Gal A.A. (2010). Granulomatous Lung Disease: An Approach to the Differential Diagnosis. Arch. Pathol. Lab. Med..

[5] Wei M., Yongjie Z., Zhuoyu Q., Biao Y., Xi J., Wei J., Tang B. (2020). Pneumonia caused by Mycobacterium tuberculosis. Microbes Infect..

[6] Septimus E.J., Awe R.J., Greenberg S.D., Raleigh J.W. (1977). Acute tuberculous pneumonia. Chest.

[7] Zhao M., Xin X.F., Hu H., Pan X.H., Lv T.F., Liu H.B., Zhang J.Y., Song Y. (2019). 18F-fluorodeoxyglucose positron emission tomography/computed tomography in the diagnosis of benign pulmonary lesions in sarcoidosis. Transl. Lung Cancer Res..

[8] Aubry M.-C. (2012). Necrotizing granulomatous inflammation: What does it mean if your special stains are negative?. Mod. Pathol..

[9] Koch M.L., Cote R.A. (1965). Comparison of Fluorescence Microscopy with Ziehl-Neelsen Stain for Demonstration of Acid-Fast Bacilli in Smear Preparations and Tissue Sections. Am. Rev. Respir. Dis..

[10] Mustafa T., Wiker H.G., Mfinanga S.G.M., Mørkve O., Sviland L. (2006). Immunohistochemistry using a Mycobacterium tuberculosis complex specific antibody for improved diagnosis of tuberculous lymphadenitis. Mod. Pathol..

[11] Huang Y.E., Lu H.I., Liu F.Y., Huang Y.J., Lin M.C., Chen C.F., Wang P.W. (2012). Solitary pulmonary nodules differentiated by dynamic F-18 FDG PET in a region with high prevalence of granulomatous disease. J. Radiat. Res..

[12] Feng M., Yang X., Ma Q., He Y. (2017). Retrospective analysis for the false positive diagnosis of PET-CT scan in lung cancer patients. Medicine.

[13] Bakheet S.M., Saleem M., Powe J., Al-Amro A., Larsson S.G., Mahassin Z. (2000). F-18 fluorodeoxyglucose chest uptake in lung inflammation and infection. Clin. Nucl. Med..

[14] Lewinsohn D.M., Leonard M.K., LoBue P.A., Cohn D.L., Daley C.L., Desmond E., Keane J., Lewinsohn D.A., Loeffler A.M., Mazurek G.H. (2017). Official American Thoracic Society/Infectious Diseases Society of America/Centers for Disease Control and Prevention Clinical Practice Guidelines: Diagnosis of Tuberculosis in Adults and Children. Clin. Infect. Dis..

[15] Shah K.K., Pritt B.S., Alexander M.P. (2017). Histopathologic review of granulomatous inflammation. J. Clin. Tuberc. Other Mycobact. Dis..

[16] Ohshimo S., Guzman J., Costabel U., Bonella F. (2017). Differential Diagnosis of Granulomatous Lung Disease: Clues and Pitfalls. Eur. Respir. Rev..

[17] To K., Cao R., Yegiazaryan A., Owens J., Venketaraman V. (2020). General Overview of Nontuberculous Mycobacteria Opportunistic Pathogens: Mycobacterium avium and Mycobacterium abscessus. J. Clin. Med..

[18] Ulbright T.M., Katzenstein A.L. (1980). Solitary necrotizing granulomas of the lung: Differentiating features and etiology. Am. J. Surg. Pathol..

[19] Silva Miranda M., Breiman A., Allain S., Deknuydt F., Altare F. (2012). The tuberculous granuloma: An unsuccessful host defence mechanism providing a safety shelter for the bacteria?. Clin. Dev. Immunol..

[20] Krishna M., Gole S.G. (2017). Comparison of Conventional Ziehl-Neelsen Method of Acid Fast Bacilli with Modified Bleach Method in Tuberculous Lymphadenitis. J. Cytol..

[21] Caulfield A.J., Wengenack N.L. (2016). Diagnosis of active tuberculosis disease: From microscopy to molecular techniques. J. Clin. Tuberc. Other Mycobact. Dis..

[22] Attorri S., Dunbar S., Clarridge J.E. (2000). Assessment of Morphology for Rapid Presumptive Identification of *Mycobacterium tuberculosis* and *Mycobacterium kansasii*. J. Clin. Microbiol..

[23] Sibley C.D., Peirano G., Church D.L. (2012). Molecular methods for pathogen and microbial community detection and characterization: Current and potential application in diagnostic microbiology. Infect. Genet. Evol..

[24] Marchetti G., Gori A., Catozzi L., Vago L., Nebuloni M., Rossi M.C., Esposti A.D., Bandera A., Franzetti F. (1998). Evaluation of PCR in detection of Mycobacterium tuberculosis from formalin-fixed, paraffin-embedded tissues: Comparison of four amplification assays. J. Clin. Microbiol..

[25] Salian N.V., Rish J.A., Eisenach K.D., Cave M.D., Bates J.H. (1998). Polymerase Chain Reaction to Detect Mycobacterium tuberculosis in Histologic Specimens. Am. J. Respir. Crit. Care Med..

